# A Modified Subpulse SAR Processing Procedure Based on the Range-Doppler Algorithm for Synthetic Wideband Waveforms

**DOI:** 10.3390/s8128224

**Published:** 2008-12-11

**Authors:** Byoung-Gyun Lim, Jea-Choon Woo, Hee-Young Lee, Young-Soo Kim

**Affiliations:** 1 Division of Electrical and Computer Engineering, Pohang University of Science and Technology, Pohang, Gyungbuk, 790-784, South Korea; E-Mails: digidev@postech.ac.kr (J. C. W); ysk@postech.ac.kr (Y. S. K.); 2 Agency for Defense Development, Daejeon, South Korea; E-Mail: youngone@add.re.kr (H. Y. L.)

**Keywords:** Synthetic Aperture Radar (SAR), Range-Doppler Algorithm (RDA), Synthetic Wideband Waveforms (SWW)

## Abstract

Synthetic wideband waveforms (SWW) combine a stepped frequency CW waveform and a chirp signal waveform to achieve high range resolution without requiring a large bandwidth or the consequent very high sampling rate. If an efficient algorithm like the range-Doppler algorithm (RDA) is used to acquire the SAR images for synthetic wideband signals, errors occur due to approximations, so the images may not show the best possible result. This paper proposes a modified subpulse SAR processing algorithm for synthetic wideband signals which is based on RDA. An experiment with an automobile-based SAR system showed that the proposed algorithm is quite accurate with a considerable improvement in resolution and quality of the obtained SAR image.

## 1. Introduction

Synthetic aperture radar (SAR) can produce high resolution two-dimensional imagery of the ground surface. The improvement in resolution is normally achieved by increasing the bandwidth, so a highresolution SAR usually transmits a wideband chirp signal. To increase the range resolution beyond the theoretical value of *c*/2*B*, where *c* is signal propagation speed and *B* is the chirp bandwidth, synthetic waveforms using a burst of narrowband signals have been suggested [1-3]. These waveforms combine the advantages of a stepped-frequency continuous wave (SF-CW) waveform and a chirp signal waveform without requiring an unrealistically high sampling rate. Such narrowband pulse sequences have many names, including a synthetic wideband signal [4], synthetic bandwidth [2, 5, 6], a stepped chirp signal [7], a stepped frequency train [3, 8, 9], and a frequency-jumped burst [10]. The papers and reports on synthetic wideband waveforms (SWW) have mainly dealt with methods to implement such waveforms [2, 5], and signal processing techniques to reduce the sidelobes and grating lobes [3, 8, 9]. Several methods including nonlinear stepping, linear windowing and spatial variant apodization have also been suggested [4, 7]. However, few studies have reported the actual quality of the SAR images acquired using these synthetic wideband waveforms.

The range migration algorithm (RMA) can properly focus a SAR signal without approximations. Alternative methods to avoid this computationally demanding algorithm are the range-Doppler algorithm (RDA) and the chirp scaling algorithm (CSA). The approximations used in RDA and CSA are well-known [11]. These algorithms have also been modified by various types of secondary range compression (SRC) and range cell migration compensation (RCMC) techniques to compensate for the cross coupling caused by high squint angle or wide aperture. However, relatively little attention has been given to the problem of large bandwidth. The purpose of this paper is to present a modified subpulse SAR processing algorithm for synthetic wideband signals to produce high resolution imagery efficiently and accurately using RDA which is easy to implement and computationally efficient. Unlike the conventional methods, our method processes each subpulse composing the large bandwidth separately using the corresponding carrier frequencies before they are stitched together. The proposed algorithm was quite effective in realistic experiments.

## 2. Synthetic Wideband Waveform Modeling

**Figure 1.**
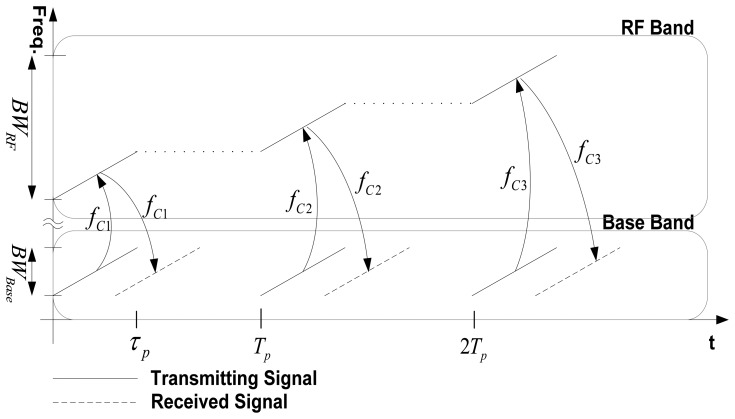
The Frequency up-down scheme of the synthetic wideband signals. *T_p_* is pulse period, *τ_p_* is pulse width, and *f_C1_, f_C2_, f_C3_*, are carrier frequencies.

The received signals from narrow subpulses can be combined to form a single pulse with a wide synthetic bandwidth, as if one pulse had been received [2, 5]. Then, pulse compression with a suitable reference function obtains a range profile with fine resolution and a high peak-to-sidelobe ratio (PSLR). This approach applies a frequency up-down scheme to the synthetic wideband signal to the bandwidth three times in the time-frequency domain (Figure 1). Baseband signals are upconverted to different RF bands and transmitted. Then the received signals are downconverted to the same baseband. By selecting different carrier frequencies, the total RF bandwidth can be extended to three times the baseband bandwidth.

The processing steps necessary to synthesize a wideband pulse from the received narrowband subpulses are: 1) upsampling; 2) frequency shift; 3) phase correction; 4) time shift; and 5) merging of the corrected subpulses.

Because the narrowband subpulses are naturally sampled at a lower rate than the desired wideband signal, they must be upsampled using zero padding in the frequency domain before combining. Then, because all upsampled narrowband pulses are at baseband, they must be shifted to the proper spectrum positions in the frequency domain. A frequency shift of *f*_Δ_ in frequency domain can be achieved by multiplying the phase term exp (2π*f*_Δ_t) in the time domain. Also, the phase of the wideband pulse must be continuous at the narrowband pulse boundaries, and a phase correction term must be added to each subpulse. Finally, before combining the individual pulses, they must be shifted in the time domain; the resultant final data is the superposition of the corrected subpulses in the range direction.

## 3. Modified RDA Procedure for Synthetic Wideband Signals

**Figure 2.**
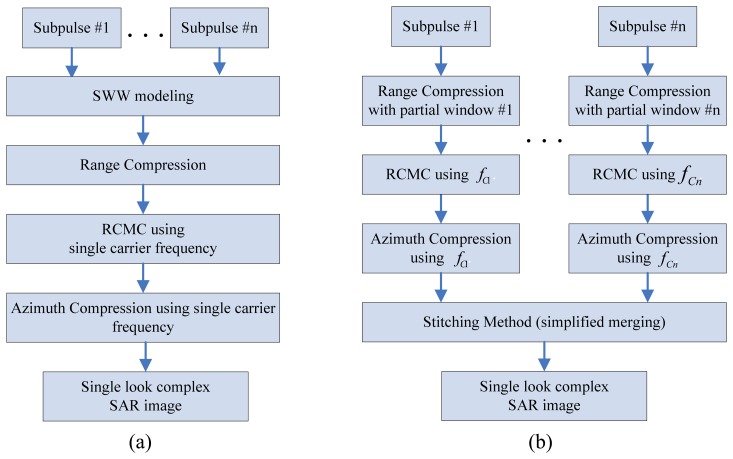
SAR processing algorithm: (a) Conventional algorithm; (b) modified RDA.

SAR images for a synthetic wideband waveform can be obtained using a conventional RDA: range compression is performed by a convolution with a single wideband reference signal in the range direction, and then RCMC is performed in the range-Doppler domain (Figure 2a). Finally, the corrected data is compressed with the azimuth reference signal using one carrier frequency. However, the quality of the SAR images obtained by the conventional RDA method is somewhat lower than expected by the synthetic wideband signal. As will be shown later, this is because each narrowband subpulse has a different carrier frequency term and therefore needs a different RCMC and azimuth compression. As the bandwidth of the SWW becomes larger for higher resolution, the effect becomes more serious. Conventional RDA for SWW normally does not consider the effect of this carrier frequency factor when the subpulses are synthesized. This paper proposes a modified RDA procedure in an attempt to improve the quality of the SAR images using synthetic wideband signals.

Our proposed procedure (Figure 2b) conducts the range compression with a partial window suitable for each subpulse and then performs the RCMC and azimuth compression after considering the carrier frequencies individually. Finally, the spectra of each set of compressed data are combined using the stitching method. The algorithm is described below in detail.

### 3.1. Range Compression with Partial Windowing

A conventional SAR processor performs range compression with a matched filtering: a range FFT is performed and multiplied with a matched filter response, then a range IFFT is performed to complete the range compression. Range compression for a synthetic wideband signal is not much different from that of the conventional single chirp signal. Because all received narrowband pulses are at baseband, the range compression is performed by matched filtering using the same reference signal (*H_r_*). The received signal *s_base_* at baseband can be expressed by range time (*t*) and azimuth position (*η*) [11].

(1) $$s_{\text{base}}(t,\eta ,n)=w_{r}(t-\tau_{n})\cdot w_{a}(\eta )\cdot \exp\left\{j\pi M{\left(t-\tau_{\eta }\right)}^{2}-j2\pi f_{\text{Cn}}\tau_{\eta }\right\}$$

where $w_{r}\left(=\text{rect}\left(\frac{t}{\tau_{p}}\right)\right)$ is the range envelope, *τ_p_* is the pulse width, $w_{a}\left(\approx \sin c^{2}\left(\frac{0.886.\theta (\eta )}{\theta_{bw}}\right)\right)$ is the azimuth envelope determined by the antenna beam pattern, *θ_bw_* is the azimuth beam width, and *θ (η)* is the angle measured from the boresight in the slant range plane. Also, *M* is the chirp rate, *f_Cn_* is the n-th carrier frequency, *τ_η_* (=2*R* (*η*)/*c*) is the time delay, and *R* (*η*) is the distance from the platform to the target. For simplicity, zero squint angle is assumed, and variation in the signal amplitude is neglected. As squint angle decreases, the cross coupling between the range and azimuth becomes weaker, so applying a rough SRC method implemented with range compression should be sufficient to correct the misfocusing caused by this coupling.

**Figure 3.**
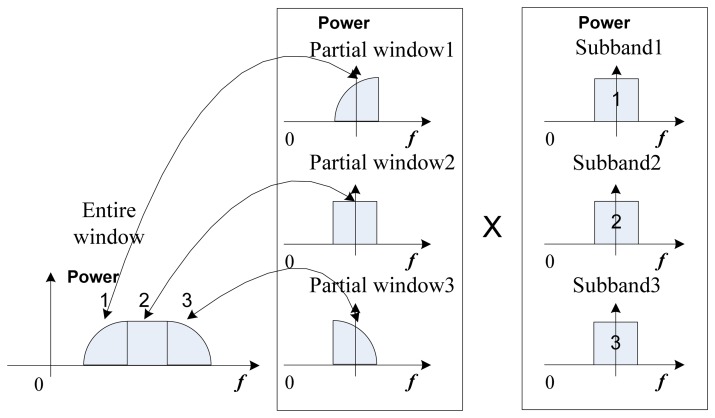
The partial windowing.

The resulting PSLR is -13dB when the envelope of the spectrum is approximately rectangular. This level of PSLR in the range direction is usually considered to be too high, and a smoothing window can be applied to the matched filter response in the frequency domain. However, the proposed algorithm (Figure 2b) processes the subpulses individually. Therefore a single window covering the entire synthetic bandwidth is not applied as in the conventional windowing technique, and a partial windowing is proposed here (Figure 3).

First, the entire window is split into *N* (number of subbands) partial windows, and shifted to the baseband. The subbands are multiplied with the spectrum data in each subband, and then all windowed data are inverse Fourier-transformed. The reference signal *H_r_* and the range compressed signal with partial windowing *s_rc_* can be expressed as:

(2) $$H_{r}(f_{t})=\text{rect}\left(\frac{f_{t}}{M\cdot \tau_{p}}\right)\cdot \exp\left(-j\pi \frac{f_{t}^{2}}{M}\right)$$

(3) $$s_{\text{rc}}(t,\eta ,n)=\text{IFF}T_{t}\left[\text{FF}T_{t}\left\{s_{\text{bace}}\left(t,\eta ,n\right)\right\}\cdot H_{r}^{*}\cdot W_{\text{PWn}}\right]=p_{\text{rn}}\left(t-\tau_{\eta }\right)\cdot w_{a}(\eta )\cdot \exp(-j2\pi f_{\text{Cn}}\tau_{\eta }),$$

where *W_PWn_* is the partial windowing applied to the n-th subband. Here, the Kaiser window with *β*=2.5 is used for the entire window. The compressed pulse envelope *p_m_* is the IFFT of the partial windowing, *W_PWn_*.

### 3.2. Range Cell Migration Compensation (RCMC)

After the range compression with partial windowing, RCMC is performed by range interpolation in the range-Doppler domain. The signal after the azimuth FFT, *S_1_* is given as:

(4) $$S_{1}(t,f_{\eta },n)=p_{\text{rn}}\left(t-\frac{2R_{\text{rd}}\left(f_{\eta },n\right)}{c}\right)\cdot w_{a}(f_{\eta })\cdot \exp\left\{-j\frac{4\pi R_{0}f_{\text{Cn}}D(f_{\eta })}{c}\right\},$$

where *D* (*f_η_*) is the range migration factor given by:

(5) $$D(f_{\eta })=\sqrt{1-{\left(\frac{f_{\eta }c}{2f_{\text{Cn}}}\right)}^{2}}.$$

Also, *R_rd_*, the range cell migration (RCM) in the range envelope, is

(6) $$R_{\text{rd}}(f_{\eta },n)=\frac{R_{0}}{D(f_{\eta })}\approx R_{0}+\frac{c^{2}R_{0}f_{\eta }^{2}}{8\cdot f_{\text{cn}}^{2}}.$$

The amount of RCM, Δ*R_rd_*, is therefore

(7) $$\Delta R_{\text{rd}}(f_{\eta },n)=R_{\text{rd}}(f_{\eta },n)-R_{0}\approx \frac{c^{2}R_{0}f_{\eta }^{2}}{8\cdot f_{\text{Cn}}^{2}}.$$

This amount of RCM is range dependent. Because one of the dimensions is range time, the RDA can compensate for the range dependency of Δ*R_rd_* in the range Doppler domain. However, when the bandwidth is a considerable portion of the carrier frequency, Δ*R_rd_* is spread further due to the frequency term. Assuming that a synthetic bandwidth with 600MHz bandwidth has a center frequency of 10GHz, the difference in Δ*R_rd_* between the start and the end frequency is about 12%. Such a difference increases as the synthetic bandwidth increases. If the RCMC is performed only for one carrier frequency when the synthetic bandwidth is not negligible, then the point target response will be dispersed to several range bins and the SAR image will have lower resolution than expected from the bandwidth. However, the proposed algorithm performs the RCMC for the carrier frequency of each subpulse individually, so an SAR image with near ideal resolution can be obtained. The RCM-compensated signal, *S*_2_ can be represented as

(8) $$S_{2}(t,f_{\eta },n)=p_{\text{rn}}\left(t-\frac{2R_{0}}{c}\right)\cdot w_{a}(f_{\eta })\cdot \exp\left\{-j\frac{4\pi R_{0}f_{\text{cn}}D(f_{\eta })}{c}\right\}.$$

### 3.3. Azimuth Compression

The azimuth compression (Figure 2b) is performed by multiplying each windowed subband dataset by the conjugated Fourier-transformed azimuth reference signal for the corresponding carrier frequency (*f_Cn_*), and a single window in the azimuth direction is applied. The azimuth reference *H_az_* and the azimuth compressed signal *s_ac_* are:

(9) $$H_{\text{az}}(f_{\eta },n)=\exp\left(-j\frac{4\pi R_{0}f_{\text{Cn}}D(f_{\eta })}{c}\right),$$

(10) $$s_{\text{ac}}(t,\eta ,n)=\text{IFF}T_{\eta }\left[S_{2}(t,f_{\eta },n)\cdot H_{\text{az}}^{*}(f_{\eta },n)\cdot W_{\text{az}}\right]=p_{\text{rn}}\left(t-\frac{2R_{0}}{c}\right)\cdot p_{a}(\eta ),$$

where *p_a_* the amplitude of the azimuth impulse response, is a sinc-like function similar to *p_m_*, *W_az_* is the azimuth window whose width is equal to the azimuthal pattern.

### 3.4. Stitching

Finally, the azimuth compressed data for each subpulse are simply stitched together. They must be upsampled first using zero padding in the frequency domain before combining (Section 2). Because all upsampled narrowband pulses are at baseband, they must be shifted to proper spectrum positions in the frequency domain. The quadratic phases caused by chirp rate and carrier frequency are completely removed by range and azimuth matched filtering of each subpulse, so no additional phase correction is necessary. However, if relative motion occurs between the antenna and the target, the phase correction in [5] should be considered.

The final operation needed is simply to sum all spectrum data in the range direction. However, when the frequency step is smaller than the bandwidth of the baseband signal, there is a problem with overlap. Due to the spectral discontinuity, undesired sidelobes and grating-lobes are generated. Also, abrupt phase changes at the boundary between the adjacent spectra can deteriorate the quality of the SAR image can be deteriorated. This problem can be avoided by applying a raised cosine filter for the range spectrum [12]. Finally, the final 2-dimensional SAR image can be expressed as:

(11) $$s_{\text{final}}(t,n)={\text{IFFT}}_{t}\left[\sum_{n=1}^{N}S_{\text{ac}}(f_{t}-(n-1)f_{\Delta },\eta ,n)\cdot H_{\text{rcf}}(f_{t})\right]$$

where *H_rcf_* is the raised cosine filter.

## 4. Experimental Results

To verify the performance of the proposed algorithm, several experiments were conducted using the automobile-based SAR (AutoSAR) system designed by Pohang University of Science and Technology (POSTECH) [13] (Table 1).

### 4.1. Single Point Target

**Figure 4.**
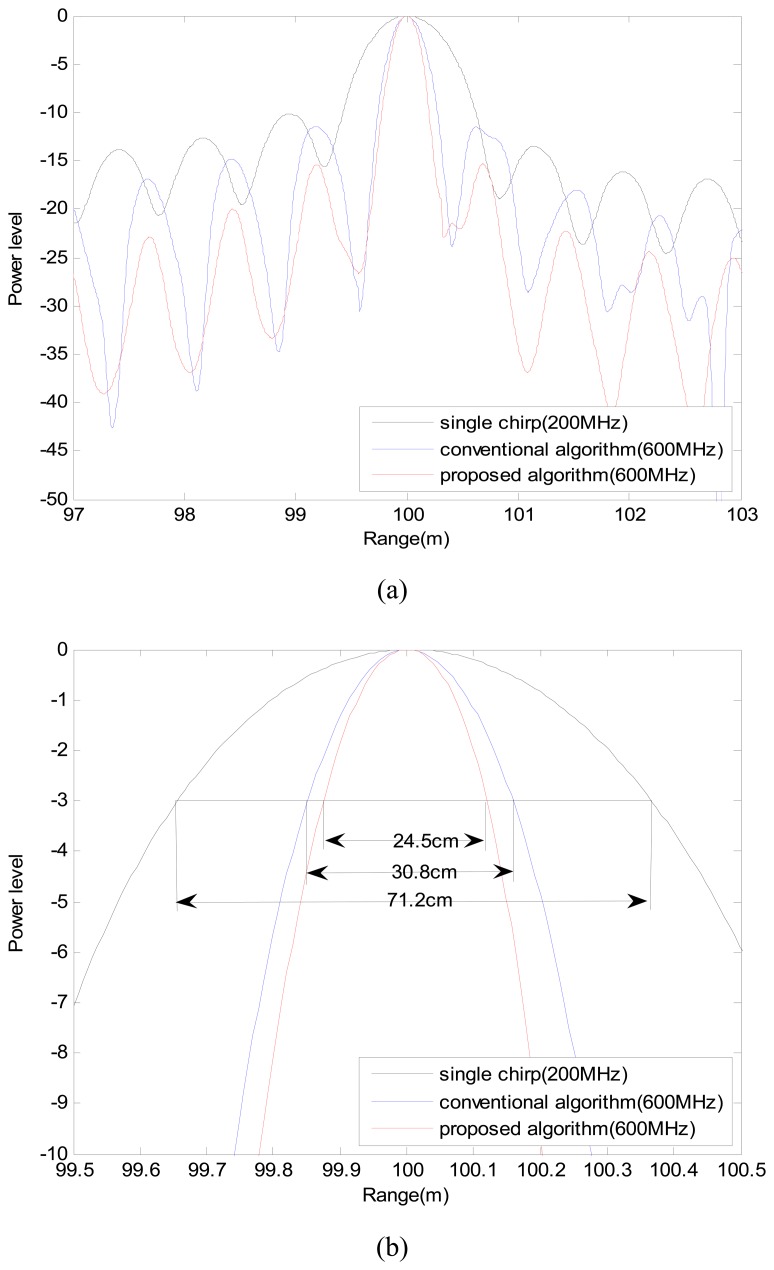

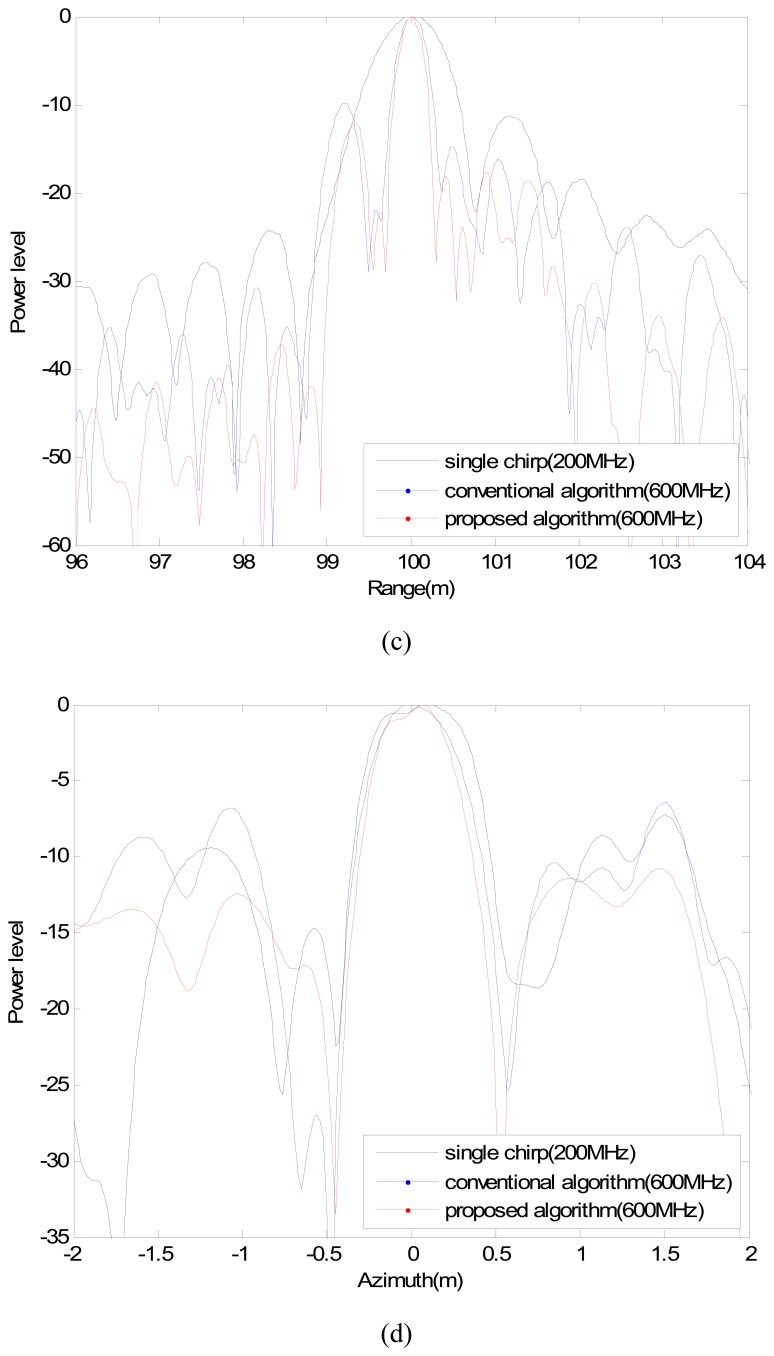
Simulation and experimental results for a point target. (a) Range profiles using simulation, (b) mainlobe comparison of simulated data, (c) measured range profiles, (d) measured azimuth profiles

To quantify the range resolution improvement of the proposed algorithms, a simulation was performed for the case of an ideal point scatterer located at a range of 100 m. The parameters of the simulations were chosen to be same as those for the AutoSAR system. The range profiles acquired by three different algorithms near the center of the point scatterer (Figure 4a) show that when the bandwidth of the chirp was expanded three times by the synthetic wideband signal, the resolution obtained by the conventional SAR algorithm was improved by only about 2.3 times (Figure 4b). In contrast, the proposed algorithm improved the resolution by almost three times, close to the theoretical value of 0.886*c*/2*B* = 22 cm.

Also, quantification of the resolution improvement was performed by measuring the resolution in the SAR images of a trihedral corner reflector located at a range of 100 m, using a single chirp of 200 MHz bandwidth, together with additional profiles using the SWW of 600 MHz bandwidth, one with the conventional algorithm and another with the proposed algorithm.

Both range (Figure 4c) and azimuth (Figure 4d) profiles obtained using the proposed algorithm show better sidelobe performance than those profiles using the conventional method. This improvement occurs because the azimuth compression of each subpulse was performed using the azimuth reference signal with a different carrier frequency (Section 3). The same point can be made with regard to the range resolution at the target center. The oversampled range resolution was 69.0 cm for a single chirp (200 MHz), 30.4 cm for an SWW (600 MHz) processed with the conventional algorithm, and 24.3 cm for a synthetic wideband signal (600 MHz) processed with the proposed algorithm. These results are quite close to the simulation results (Figure 4 b).

### 4.2. Distributed Targets

**Figure 5.**
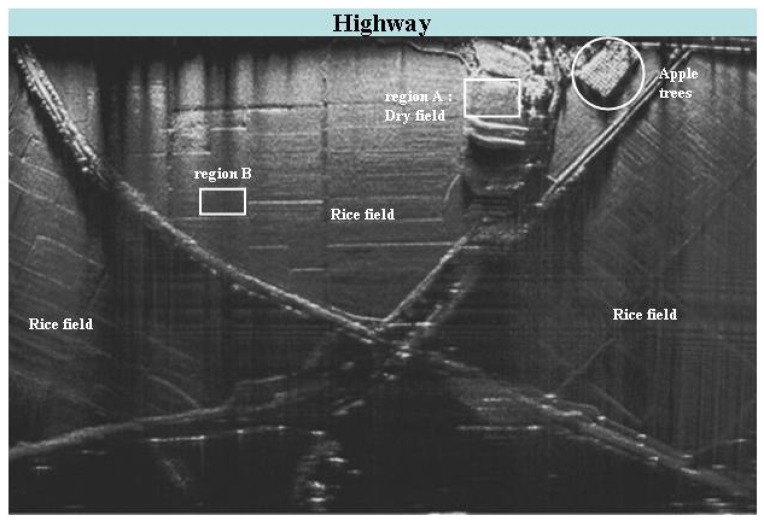
SAR image of a rural area obtained by AutoSAR [13]. The size of imaged area is about 800 m × 600 m.

A SAR image (Figure 5) of a typical rural area near Pohang, Korea was obtained using a single chirp of 200 MHz. We obtained SAR raw data from one side while driving on the straight highway (located at the top of Figure 5).

**Figure 6.**
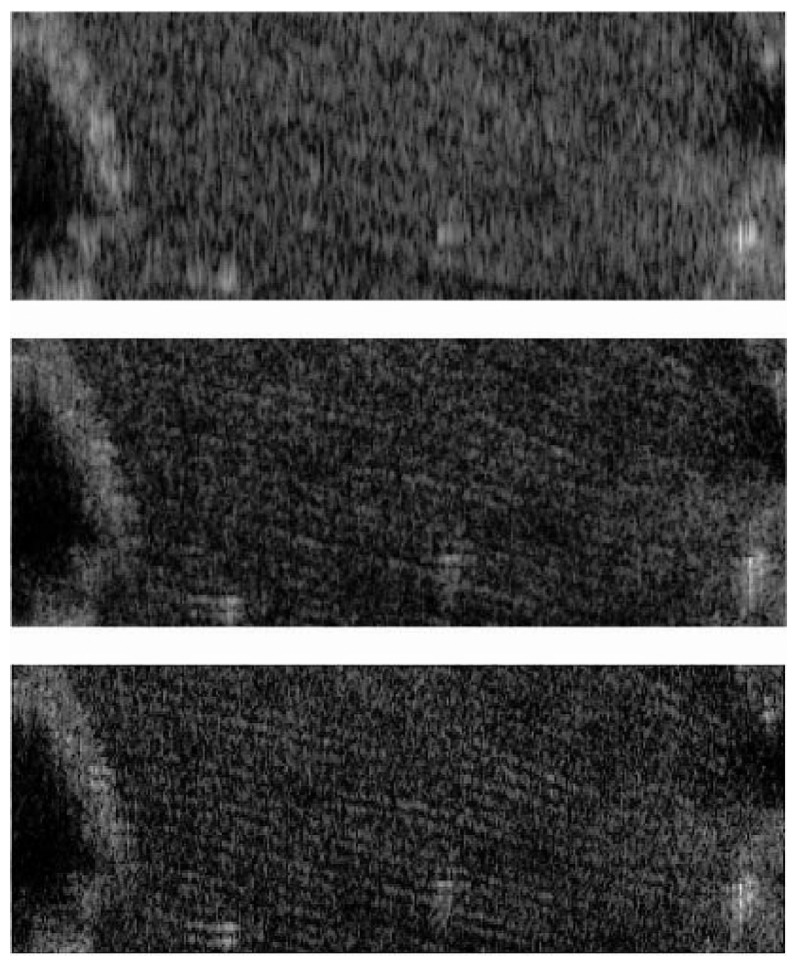
An expanded image of region A. Single chirp 200 MHz (top), SWW (600 MHz) with conventional algorithm (center), SWW (600 MHz) with proposed algorithm (bottom). The size of each imaged area is 70 m × 20 m.

Region A is in dry field, which was selected to visualize the effect of the range resolution improvement over textured distributed targets. The expanded images of region A, using a single chirp (200 MHz), an SWW (600 MHz) processed with the conventional algorithm, and a synthetic wideband signals (600 MHz) processed with the proposed algorithm, (Figure 6) demonstrate that the proposed algorithm shows the furrows of the dry field much more clearly and in more detail than the conventional algorithm.

Another method of measuring the range resolution improvement is to calculate the resolution of the speckle over a homogeneous area [12]. A spatial autocorrelation (SA), *ρ* (Δ*k*), is:

(12) ρ(Δk)=|E[u(k)⋅u(k+Δk)]|,

where *k* is the pixel under consideration, Δ*k* is the range distance from pixel *k*, *u* is the complex image value, and *E* represents the expectation operator. When resolution is perfect, this function approaches a δ–function. Over a homogeneous area, region B of Figure 5, the SA was calculated for the single chirp and SWW with two different algorithms. A summary of simulation results, measurement results for a point target, and the SA results for a distributed target (Table 2) demonstrates that the proposed algorithm shows better performance than the conventional algorithm.

## 5. Conclusions

Many papers have dealt with synthetic wideband waveforms, but most have emphasized methods to implement SWW or methods to reduce the sidelobes and grating lobes. Also, they tend to use conventional SAR processing algorithms without much attention to the errors, which may not be negligible when a very wide bandwidth is used. This paper proposes a modified RDA procedure in an attempt to improve the quality of SAR images from SWW. Experiments with an automobile-based SAR system showed that the proposed algorithm is quite accurate in processing the synthetic wideband signals, with a resolution improvement of 20 to 30% compared to the conventional SAR processing algorithm. Moreover, if parallel processing is possible, subpulses can be processed independently and merged to obtain a high quality SAR image much more efficiently.

## Figures and Tables

**Table 1.** The specification of AutoSAR

**Parameters**	**Values**

Pulse period, *T_p_*	10 *μs*
Pulse width, *τ_p_*	4 *μs*
Carrier frequencies, *f_Cn_*	9.45, 9.65, 9.85 GHz
RF Bandwidth	up to 600 MHz
Baseband Bandwidth	200 MHz
Sampling frequency	500 MHz
Beamwidth (range, azimuthal)	10°, 5°
Azimuth sampling interval	3 cm

**Table 2.** The summary of the range resolution measured by several cases.

**Methods**	**Range resolution (cm)**			

	**Theory**	**Simulation**	**Measurement**	

			**Point target**	**Distributed target (SA)**

Single chirp (200 MHz)	66.5	71.2	69.0	68.4
Conventional algorithm (600 MHz)	22.2	30.8	30.4	31.5
Proposed algorithm (600 MHz)	22.2	24.5	24.3	22.4
